# Manufacturing and *in vivo *inner ear visualization of MRI traceable liposome nanoparticles encapsulating gadolinium

**DOI:** 10.1186/1477-3155-8-32

**Published:** 2010-12-18

**Authors:** Jing Zou, Rohit Sood, Sanjeev Ranjan, Dennis Poe, Usama A Ramadan, Paavo KJ Kinnunen, Ilmari Pyykkö

**Affiliations:** 1 Department of Otolaryngology, University of Tampere, Medical School, Tampere, Finland; 2 Helsinki Biophysics and Biomembrane Group, Department of Biomedical Engineering and Computational Sciences, Aalto University, Helsinki, Finland; 3 Experimental MRI Laboratory, Department of Neurology, Helsinki University Central Hospital, Helsinki, Finland

## Abstract

**Background:**

Treatment of inner ear diseases remains a problem because of limited passage through the blood-inner ear barriers and lack of control with the delivery of treatment agents by intravenous or oral administration. As a minimally-invasive approach, intratympanic delivery of multifunctional nanoparticles (MFNPs) carrying genes or drugs to the inner ear is a future therapy for treating inner ear diseases, including sensorineural hearing loss (SNHL) and Meniere's disease. In an attempt to track the dynamics and distribution of nanoparticles *in vivo*, here we describe manufacturing MRI traceable liposome nanoparticles by encapsulating gadolinium-tetra-azacyclo-dodecane-tetra-acetic acid (Gd-DOTA) (abbreviated as LPS+Gd-DOTA) and their distribution in the inner ear after either intratympanic or intracochlear administration.

**Results:**

Measurements of relaxivities (r1 and r2) showed that LPS+Gd-DOTA had efficient visible signal characteristics for MRI. *In vivo *studies demonstrated that LPS+Gd-DOTA with 130 nm size were efficiently taken up by the inner ear at 3 h after transtympanic injection and disappeared after 24 h. With intracochlear injection, LPS+Gd-DOTA were visualized to distribute throughout the inner ear, including the cochlea and vestibule with fast dynamics depending on the status of the perilymph circulation.

**Conclusion:**

Novel LPS+Gd-DOTA were visible by MRI in the inner ear *in vivo *demonstrating transport from the middle ear to the inner ear and with dynamics that correlated to the status of the perilymph circulation.

## Background

**Figure 1:**
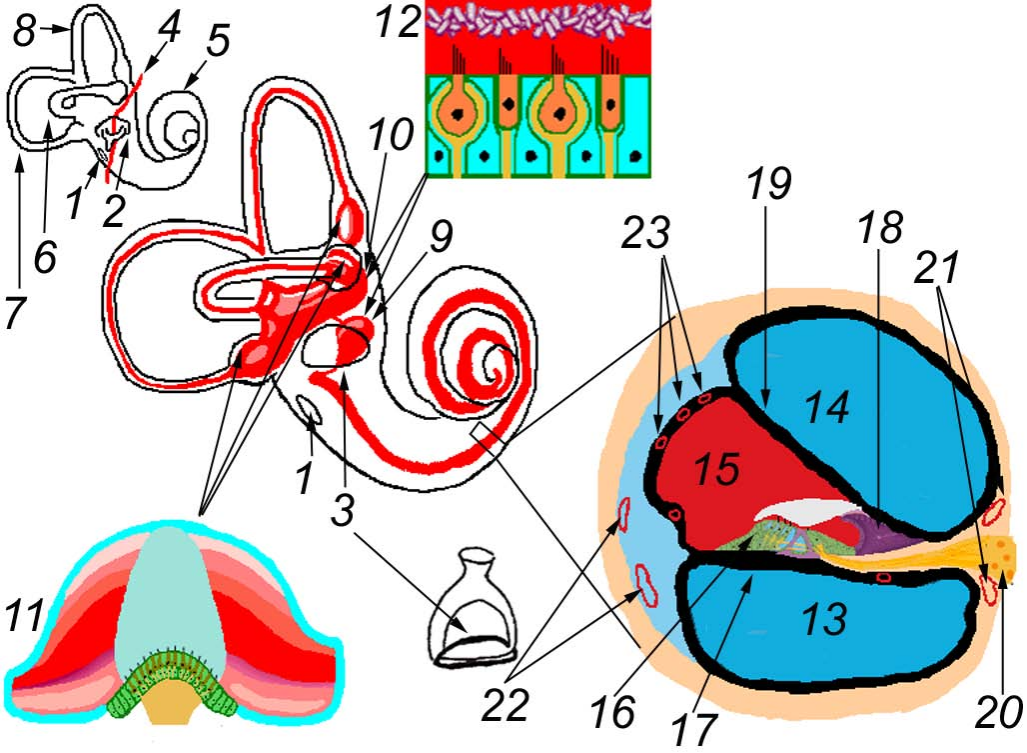
**Illustration of rat inner ear anatomy and transportation routes of substances to be gained in the inner ear after different administrations**. After intratympanic delivery, agents may enter the inner ear through the round window membrane (1) and annular ligament (2), which surrounds the stapes footplate within the oval window and comprises the stapediovestibular joint (3). Stapedial artery (4) is a prominant structure in the rat middle ear, which runs through the obturator foramen of the stapes and inferior to the round window niche. The inner ear is composed of the cochlea (5) and vestibule. The vestibule contains three semicircular canals including the lateral (6), posterior (7) and superior canals (8), and the saccule (9) and utricle (10). The crista (11), which is located in each ampulla, is the sensory structure of the semicircular canals. The macula (12) is the sensory structure of both the saccule and utricle. The cochlea contains three chambers, which are the scala tympani (13), the scala vestibuli (14), and the scala media (15). The scala tympani and vestibuli are filled by perilymph (high sodium-containing fluid). The scala media is filled with endolymph (high potassium-containing fluid) and also contains the sensory structure, the organ of Corti (16). The basilar membrane (17), which extends from the osseous spiral lamina (18), separates the scala media from scala tympani. Reissner's membrane (19) separates the scala media from scala vestibuli. Spiral ganglion cells (20), which are located in the modiolus, send peripheral processes to the hair cells and central processes to the cochlear nuclei via cochlear nerve. Capillaries in the modiolus (21) and spiral ligament (22) contribute to the blood-perilymph barrier, which is the transportation pathway of agents to reach the inner ear after intravenous or oral administration. Capillaries in the stria vascularis (23) are attributable to the blood-endolymph barrier.

Treatments of inner ear diseases with traditional strategies have had limited success and the goal of sensory organ and nerve repair or regeneration has yet to be achieved. One of the greatest challenges has been the limitations to passage through the blood-inner ear barriers and uncontrollable delivery of treatment agents after either intravenous or oral administration (Figure 1) [1]. Multifunctional nanoparticles (MFNPs) are a promising new means for the delivery of gene or drug to the inner ear for the treatment of inner ear diseases including sensorineural hearing loss (SNHL) and Meniere's disease. Eight types of MFNPs are currently under development within the European Union consortium, Nanoear [2] with the intention that they can be applied with an intratympanic, minimally-invasive approach in the clinic. MFNPs can be functionalized with the application of surface ligands to improve cellular or nuclear internalization to improve the targeted delivery of therapeutic agent. In some of our preliminary studies lipid nanocapsules were shown using confocal microscopy to become distributed in different cochlear cell populations and liposome nanoparticles functionalized with TrkB ligand were internalized into cochlear cells after round window membrane permeation [3,4].

In the process of optimizing the functionalization of each type of nanoparticle, it is essential to be able to trace their passage through the cochlea. However, it is problematic to track the MFNPs in the inner ear by histological means. First, the appearance of autofluorescence from lipofuscin granules, which are found in the cochleae of humans, chinchillas, guinea pigs and rats, impairs the detection of the fluorescence signals from the MFNP targets [5-10]. Second, the inner ear is composed of fluids, soft tissue, and bone [1] (Figure 1). This results in signal loss in histological study, which is incapable of retaining the inner ear fluids. Finally, it is inconvenient to observe the dynamics of MFNPs in the inner ear in histological studies.

MRI is an excellent tool to trace MFNPs that are labeled with a contrast agent through the inner ear by detecting their signal changes in both the inner ear fluids and soft tissue *in vivo*. The critical regions of the cochlea, including the lateral wall, spiral ganglion cells, and cochlear nerve, are also demonstrated with the resolution of a 4.7 T MRI [11,12]. Gadolinium- tetra-azacyclo-dodecane-tetra-acetic acid (Gd-DOTA) was shown to be efficiently taken up into rat inner ears and detected at 60 min post-transtympanic injection with greater signal intensity seen in the scala vestibuli than in the scala tympani and no uptake into the endolymph after four hours [11]. In the present study, amphiphilic MRI-traceable liposome nanoparticles were first developed from 1,2-ditetradecanoyl-sn-glycero-3-phosphoethanolamine-N-diethylenetriaminepentaacetic acid (gadolinium salt) [DMPE-DTPA (Gd)] (LPS-DTPA-Gd). The contribution of effective Gd (water accessible Gd) to MR signal was evaluated by disassembling Gd chelate lipid nanoparticles with 5% sodium dodecyl sulfate (SDS). Finally, effective MRI traceable liposome nanoparticles were developed by encapsulating Gd-DOTA in the nanoparticles. These nanoparticles, which are abbreviated LPS+Gd-DOTA, were finally visualized in vivo in the rat inner ears with MRI.

## Results

### Contribution of effective Gd to MR signal intensity of LPS-DTPA-Gd

In phantom testing, *LPS-DTPA-Gd *showed characteristics of a T1-contrast agent, but the maximum available concentration of the nanoparticles was unable to induce enough signal intensity for *in vivo *application. Nanoparticles dissociated by 5% SDS demonstrated T1 and T2 relaxation times similar to that of Gd-DOTA with the same molar concentration of Gd (Table 1). As the ratio of Gd chelate to lipid within the material was already maximized, an alternative strategy was pursued to enhance the r1 of the nanoparticles. LPS+Gd-DOTA nanoparticles were developed by encapsulating Gd-DOTA into the aqueous core of liposomes to obtain nanoparticles better visible by MR imaging. T1 relaxation time of purified LPS+Gd-DOTA at 160 folds dilution was close to that of LPS-DTPA-Gd at 1 mM concentration while T1 relaxation time of unpurified LPS+Gd-DOTA at 320 folds dilution was still significantly shorter than both of them (Table 1). Unpurified LPS+Gd-DOTA in artifical perilymph showed r1 of 2.2 S^-1^mM^-1 ^and r2 of 4.4 S^-1^mM^-1 ^while the purified LPS+Gd-DOTA generated r1 of 0.033 S^-1^mM^-1 ^and r2 of 0.037 S^-1^mM^-1 ^in both artifical perilymph and HEPES.

**Table 1:** Relaxation times of different liposome nanoparticles at certain concentrations.

Samples	Nanoparticle Concentrations	Gd concentrations	Relaxation times (mS) (mean ± SD)	
			
			T1	T2
LPS-DTPA-Gd	1 mM	0.5 mM	1897 ± 370	273 ± 4
LPS-DTPA-Gd+5%SDS	1 mM	0.5 mM	1697 ± 380	455 ± 5
Gd-DOTA		0.5 mM	1566 ± 324	447 ± 5
pLPS+Gd-DOTA*	0.00625 mM	3.125 mM	1980 ± 11	620 ± 7
upLPS+Gd-DOTA†	0.003125 mM	1.5625 mM	184 ± 13	102 ± 1

*purified LPS+Gd-DOTA, samples were diluted 160 folds; † unpurified LPS+Gd-DOTA, samples were diluted 320 folds.

### Transportation of LPS+Gd-DOTA through the middle-inner ear barriers

**Figure 2:**
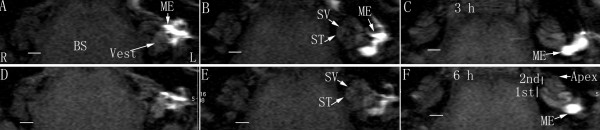
**Visualization of LPS+Gd-DOTA in the middle ear cavity and inner ear of rat after transtympanic injection using T1-weighted 2D MRI**. LPS+Gd-DOTA nanoparticles (1.0 mM LPS+Gd-DOTA containing 500 mM Gd-DOTA) were delivered to the left middle ear. At 3 h time point (A-C), they were detected in the middle ear cavity, vestibule, and cochlea. Aftert 6 h (D-F), these regions with uptake of nanoparticles showed brighter signal and the second turn and apex of the cochlea became more visible. 1st: the basal turn; 2nd: the second turn; BS: brainstem; L: left ear; ME: middle ear; R: right ear; ST: scala tympani; SV: scala vestibuli; Vest: vestibule. Particle sizes = 150 ± 20 nm. Scale bar = 1 mm.

**Figure 3:**
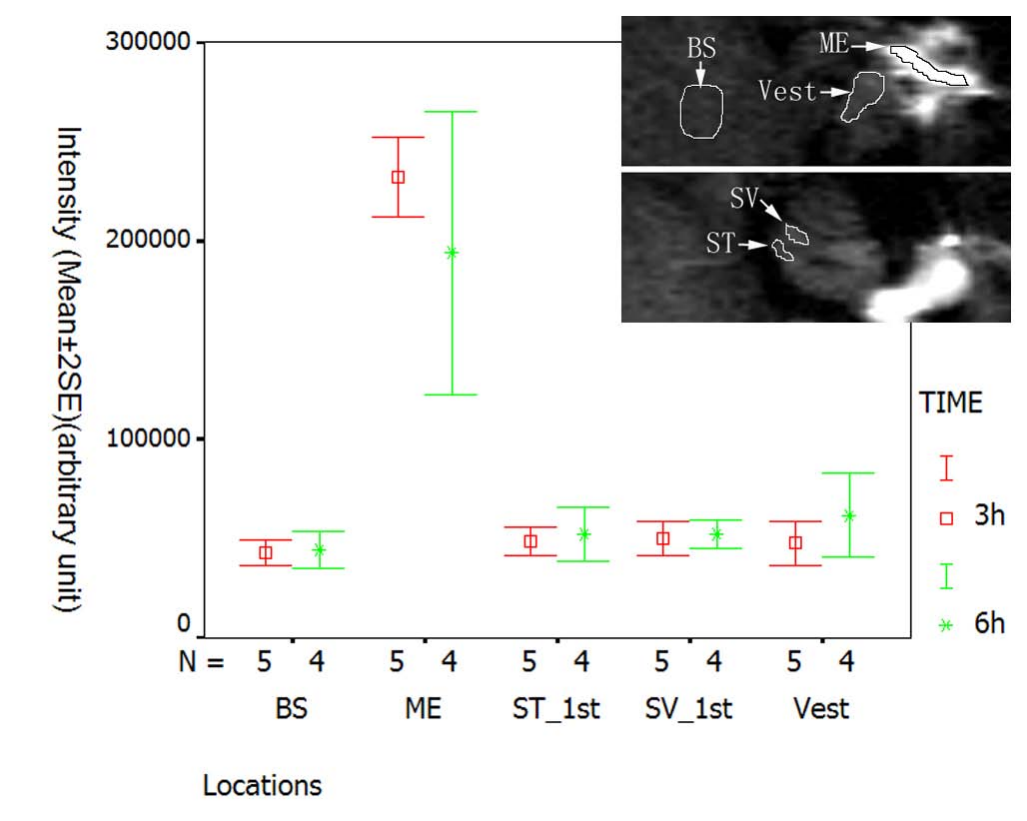
**Semi-quantification of LPS+Gd-DOTA in the middle ear cavity and inner ear of rat after transtympanic injection demonstrated by T1-weighted 2D MRI**. 1.0 mM LPS+Gd-DOTA (containing 500 mM Gd-DOTA) was administered. At 3 h post-administration, LPS+Gd-DOTA nanoparticles were efficiently detected in the perilymph, which reached 21.1% (average of the vestibule, scala vestibuli, and scala tympani) of that in the middle ear cavity. LPS+Gd-DOTA intensity decreased in the middle ear cavity while increased in the inner ear and brainstem form 3 h to 6 h after intra tympanic administration. The region of interest was shown in the images. BS: brainstem; ME: middle ear; ST: scala tympani; SV: scala vestibuli; Vest: vestibule.

**Figure 4:**
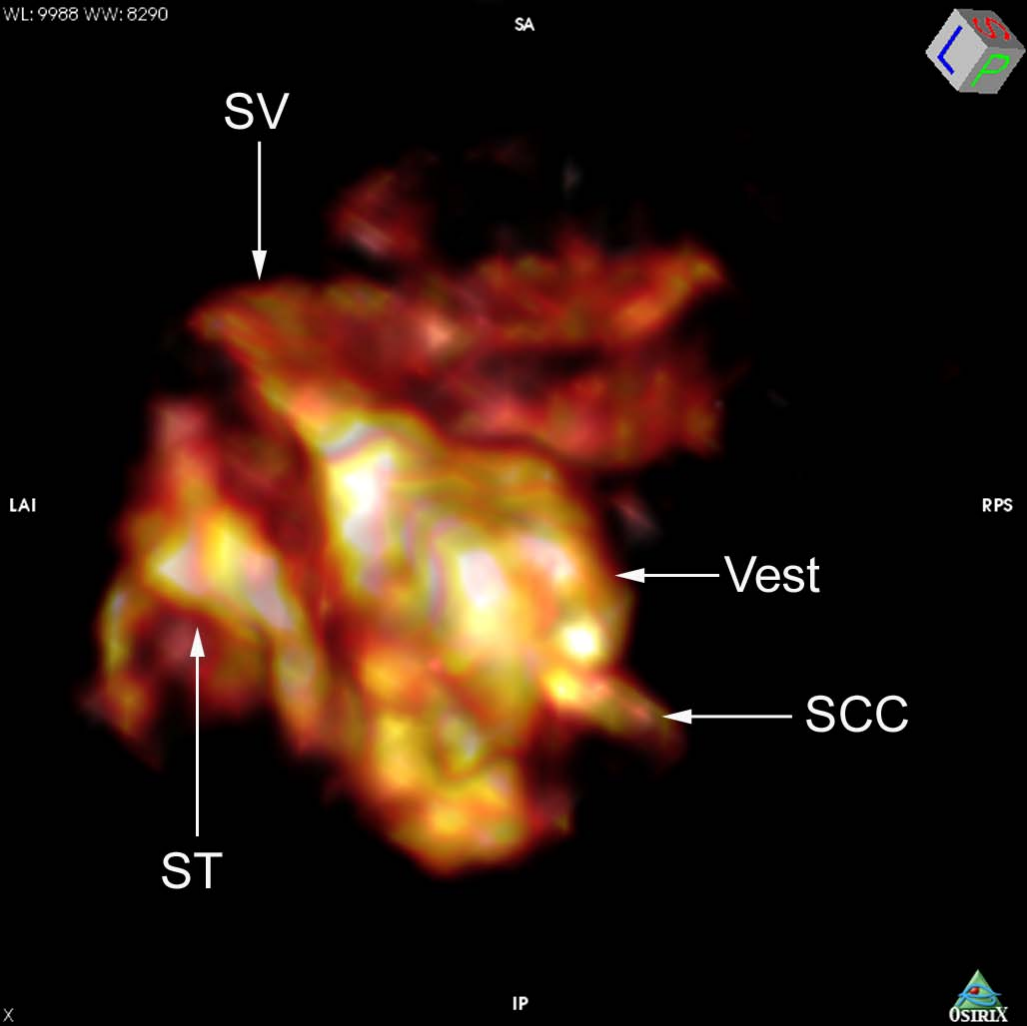
**3D rendering of T1-weighted MRI demonstrating spatial distribution of LPS+Gd-DOTA in the rat inner ear after transtympanic injection**. 1.0 mM LPS+Gd-DOTA (containing 500 mM Gd-DOTA) was administered. Abundant nanoparticles retained in the vestibule (Vest) and perilymphatic compartments of the basal lower turn. SCC: semicircular canal; ST: scala tympani; SV: scala vestibuli.

**Figure 5:**
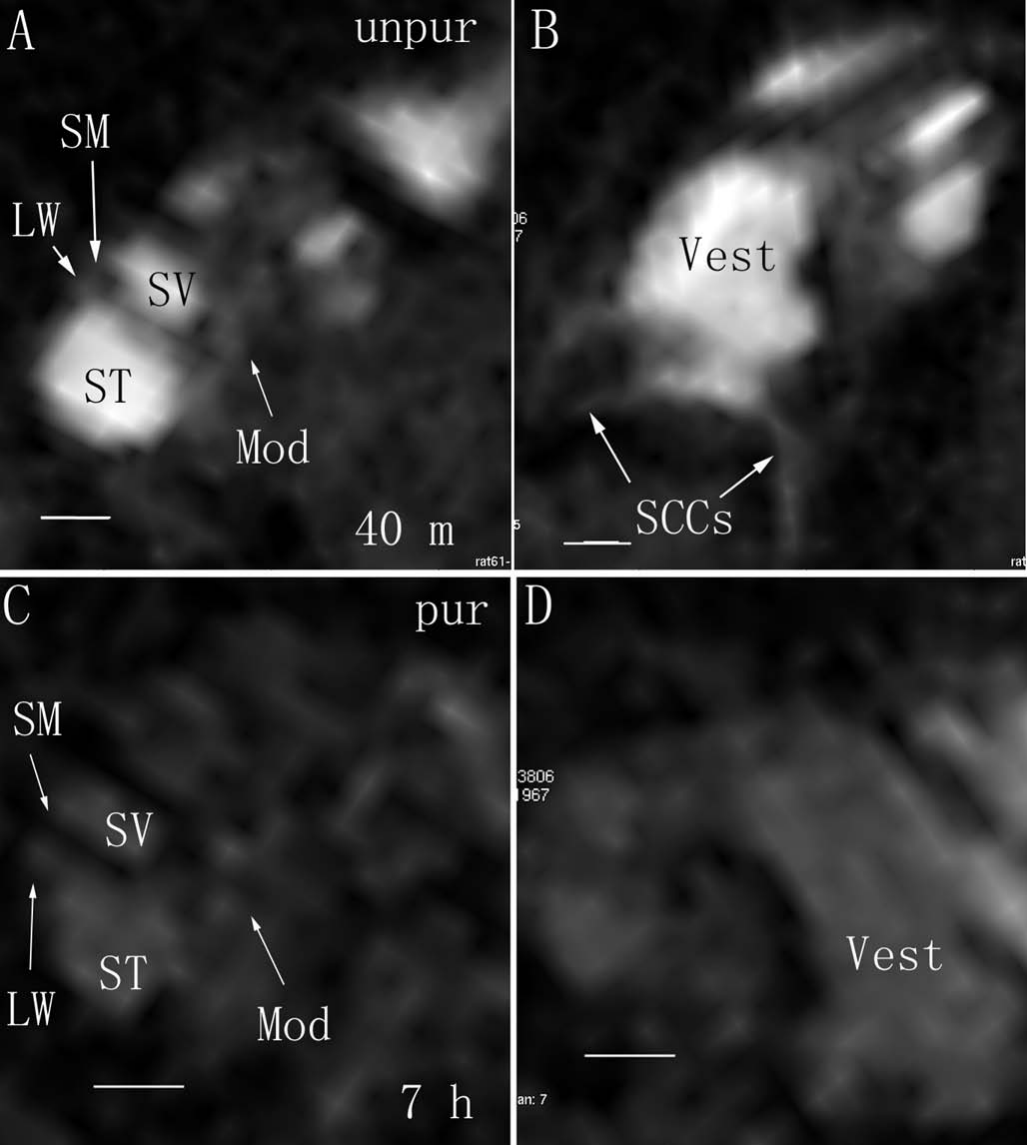
**Different uptake of unpurified and purified LPS+Gd-DOTA in rat inner ear after transtympanic injection shown by MPR single view of T1-weighted 3D MR imaging**. Unpurified LPS+Gd-DOTA were efficiently taken up in the inner ear at 40 min after transtympanic injection at a concentration of 1 mM (containing 500 mM Gd-DOTA) (A and B). The signal intensity of purified LPS+Gd-DOTA (B and C) in the inner ear was significantly lower than that of unpurified LPS+Gd-DOTA judged by vision. LW: lateral wall; Mod: modiolus; MPR: multiplanar reconstruction; SM: scala media; ST: scala tympani; SV: scala vestibuli; Vest: vestibule; SCCs: semicircular canals; unpur: unpurified LPS+Gd-DOTA; pur: purified LPS+Gd-DOTA. Particle sizes = 300 ± 20 nm. Sacle bar = 500 μm.

When applied to the middle ear cavity through transtympanic injection, 1 mM LPS+Gd-DOTA nanoparticles containing 500 mM Gd-DOTA were detected in the middle ear cavity with strong T1-weighted signal which decayed between 3 h to 6 h post administration (Figure 2 and Figure 3). In the inner ear, LPS+Gd-DOTA nanoparticles were efficiently detected in the perilymph at 3 h post-administration, which reached 21.1% (average of the vestibule, scala vestibuli, and scala tympani) of that in the middle ear cavity (Figure 2 and Figure 3). After 6 h, the singal intensity in the inner ear slightly increased though statistic significance was not achieved indicating that more LPS+Gd-DOTA nanoparticles were gained in the inner ear. 3D rendering image showed that abundant nanoparticles retained in the vestibule and perilymphatic compartments of the basal lower turn (Figure 4). Within the cochlea, additional nanoparticles had diffused into the higher turns of the cochlea, which made the perilymph in the second turn and apex more visible. The signal intensity in the brainstem was also slightly enhanced at 6 h in comparison to 3 h though without statistic significance (Figure 2 and Figure 3). An important phenomenon was noticed, in that the uptake of purified LPS+Gd-DOTA in the inner ear was influenced by the posture of the animal after transtympanic injection. In order to be able to scan immediately following injection, two rats were placed into the MRI machine in the prone position and LPS+Gd-DOTA nanoparticles were not detected in their inner ears at 3 hours post-administration. However, when rats were laid in the lateral position with the exposed ear upwards for about 2.5 h before scanning, obvious uptake of LPS+Gd-DOTA occurred in the inner ear at 3 h post-administration (Figure 2). LPS+Gd-DOTA nanoparticles were not detectable in the inner ear after neither 24 h nor 48 h in either group. Unpurified LPS+Gd-DOTA nanoparticles were efficiently detected in the perilymph of the inner ear at 40 min post-transtympanic injection (Figure 5).

**Figure 6:**
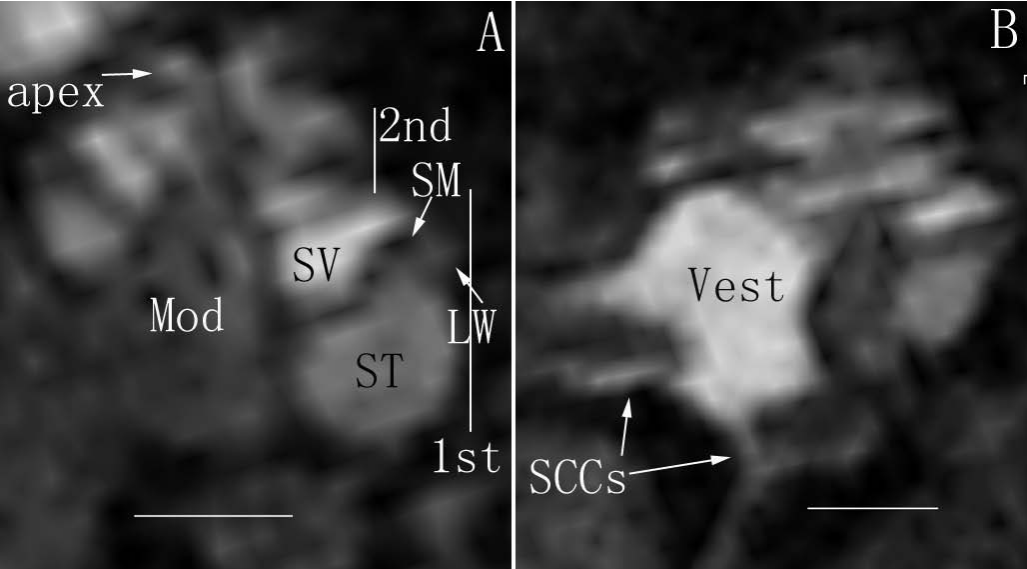
**MPR single view of T1-weighted 3D MR imaging of the rat inner ear after transtympanic injection of Gd-DOTA**. Gd-DOTA distributed universally in the cochlea and vestibule at 3 h post-transtympanic injection. LW: lateral wall; Mod: modiolus; MPR: multiplanar reconstruction; SCCs: semicircular canals; SM: scala media; ST: scala tympani; SV: scala vestibuli; Vest: vestibule. Scale bar = 1 mm.

**Figure 7:**
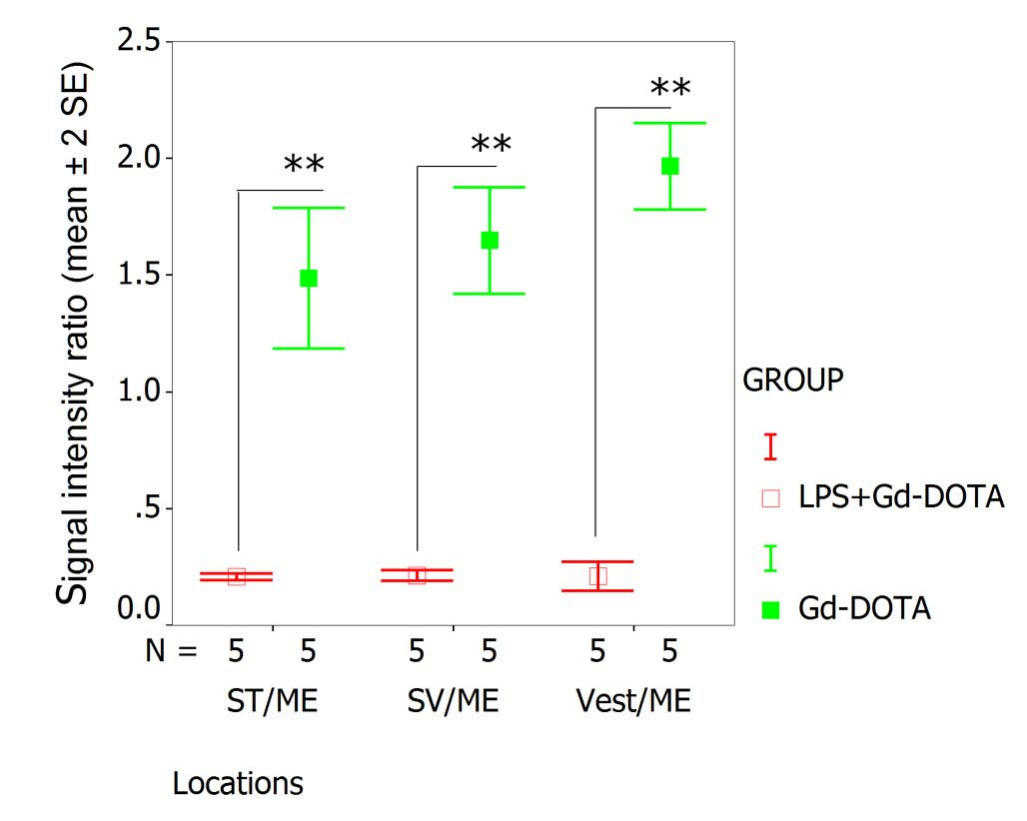
**Semi-quantitative comparison of signal ratios in the perilymphatic compartments over the middle ear cavity between LPS+Gd-DOTA and Gd-DOTA acquired by T1-weighted 2D MRI**. LPS+Gd-DOTA was transported less effectively than Gd-DOTA alone through the middle-inner ear barriers (**p < 0.01, student t-test). ME: middle ear; ST: scala tympani; SV: scala vestibuli; Vest: vestibule.

When Gd-DOTA (100 mM) alone was injected into the middle ear cavity, universal distribution in the inner ear was observed within 1 h (Figure 6). When same regions of interest were quantified, transport efficacy of Gd-DOTA across the middle-inner ear barriers into the inner ear compartments was significantly higher than that of LPS+Gd-DOTA at 3 h post-administration (p < 0.01, student t-test) (Figure 7).

### Distribution of LPS+Gd-DOTA in the inner ear after intracochlear injection

**Figure 8:**
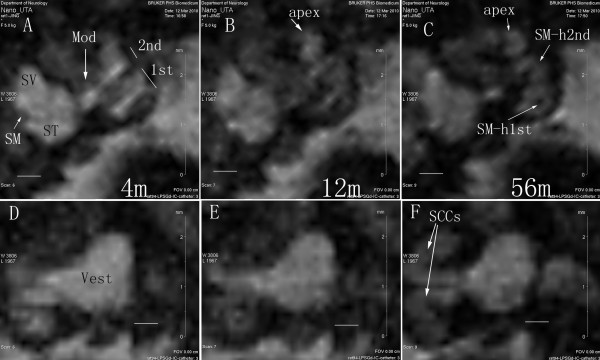
**Dynamic distribution of LPS+Gd-DOTA in the inner ear after intracochlear delivery in closed perilymphatic compartments demonstrated by MPR single view of T1-weighted 3D MRI**. 1 mM nanoparticles containing 500 mM Gd-DOTA were administered. In the cochlea, LPSG+Gd-DOTA filled SM at the beginning, but were cleaned from SM after 56 m (A, B, and C). In the vestibule, LPS+Gd-DOTA retained in the proximal locations of SCCs, but distributed to the distal parts of SCCs at 56 m post-injection (D, E, F). 1^st^: the basal turn; 2^nd^:the second turn; 4 m, 12 m, and 56 m: 4 minutes, 12 minutes, and 56 minutes; Mod: modiolus; MPR: multiplanar reconstruction; SSCs: semicircular canals; SM: the scala media; SM-h1st: SM of the higher basal turn; SM-h2nd: SM of the higher second turn; ST: the scala tympani; SV: the scala vestibuli; Vest: vestibule. Scale bar = 500 μm.

Intracochlear administration of LPS+Gd-DOTA with a volume 5 μl (1 mM LPS+Gd-DOTA containing 500 mM Gd-DOTA) in a closed perilymph circulation system through a catheter failed to produce visible signal in the inner ear. 10 μl volume of the same LPS+Gd-DOTA induced bright signal on T1-weighted images mainly in the region adjacent to the injection, that is, in the basal turn of the scala tympani, scala vestibuli, and modiolus immediately after intracochlear injection, (Figure 8). However, no further diffusion to the distal locations of the inner ear was visualized with prolonged observation time. When 50 μl was injected, using higher pressure to drive the nanoparticles, a broader distribution of LPS+Gd-DOTA in the inner ear was visualized, including the cochlea and the adjacent scala vestibuli. An abundance of LPS+Gd-DOTA nanoparticles were demonstrated in the modiolus at the level of the basal turn. In the scala vestibuli, the signal intensity was lower than in the scala tympani (Figure 8). At 56 min, LPS+Gd-DOTA nanoparticles diffused to distal sites, such as the apex of the cochlea and the semicircular canals in the vestibular organ (Figure 8).

**Figure 9:**
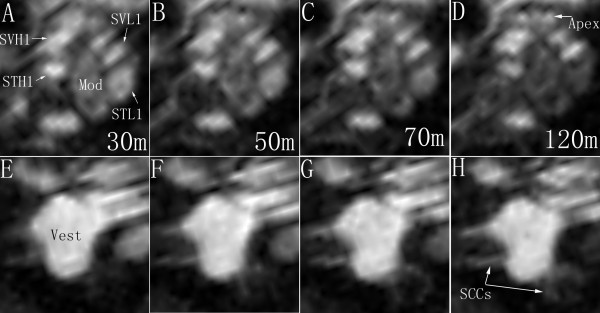
**Dynamic distribution of LPS+Gd-DOTA in the inner ear after intracochlear delivery in an open perilymphatic compartments demonstrated by MPR single view of T1-weighted 3D MRI**. 1 mM nanoparticle containing 500 mM Gd-DOTA were administered. Intracochlear dynamics was shown in A through D; intravestabular dynamics was shown in E through H. From 30 min to 120 min post-administration of LPS+Gd-DOTA, signal intensities decayed gradually in the STL1, STH1, and Mod; but retained stable in the SVL1, SVH1, and Vest. 30 m, 50 m, etc.: 30 minutes etc; Mod: modilous; MPR: multiplanar reconstruction; SSCs: semicircular canals; STH1: scala tympani of the higher basal turn; STL1: scala tympani of the lower basal turn; SVH1: scala vestibuli of the higher basal turn; SVL1: scala vestibuli of the lower basal turn; Vest: vestibule.

Intracochlear administration of LPS+Gd-DOTA nanoparticles through gelatin sponge applied to an open perilymph system, with windows created in both the scala vestibuli and scala tympani, induced highly intense bright signal in the inner ear on T1-weighted images acquired at 30 min (Figure 9). At this time point, the signal intensity in the perilymph of the vestibule was higher than in the perilymph of the cochlea, which was in turn higher than the modiolus. Homogeneous distribution of LPS+Gd-DOTA nanoparticles was observed throughout the perilymphatic compartments of the cochlea. A dynamic reduction of signal in the hook region of the scala tympani and the modiolus of the cochlea was demonstrated, which became faint at 120 min post-administration (Figure 9). In the vestibular organ, gradual enhancement occurred in the semicircular canals over 120 min while no visible change was found in the vestibule, which remained bright throughout this period (Figure 9).

## Discussion

In the present work, we demonstrated that multifunctional liposome nanoparticles, which were tagged with gadolinium, were visualized by MRI. T1 signal enhancement correlates to the amount of water accessible to the gadolinium, shown by the phenomenon that SDS treatment induced T1 relaxation time of LPS-DTPA-Gd equivalent to that of Gd-DOTA at the same concentration. It was reported that Gd-DOTA and Gd-DTPA showed similar relaxivities [13]. This suggested that each LPS-DTPA-Gd molecule became water accessible upon dissolution by SDS. Because the ratio of Gd to lipid is nonadjustable in *LPS-DTPA-Gd*, there was difficulty in generating sufficient MR signal. Alternatively, encapsulation of Gd-DOTA into the liposomes was an improved technique to increase the amount of gadolinium available for MR imaging. With this method it was possible to encapsulate 500 mM Gd into the liposomes compared to LPS-DTPA-Gd where the maximum concentration of incorporated Gd was 0.5 mM. Moreover, highly monodisperse NPs were obtained when Gd-DOTA was encapsulated into the liposomes. This is critical for quality control in pharmaceutics, and correlates with treatment efficacy. It is important to note that the gadolinium within LPS+Gd-DOTA nanoparticles is carried in the aqueous core and the shell can be manufactured to be identical to that of the treatment nanoparticles. Therefore, this MRI study is directly translatable for predicting the destination of these liposome nanoparticles in clinical use to carry genes or drugs.

Gd-DOTA-tagged liposome nanoparticles were demonstrated within the inner ears of rats *in vivo *using MRI. After intracochlear injection, nanoparticles diffused throughout the inner ear efficiently and immediately reached the modiolus, which includes the spiral ganglion cells. Therefore, the multifunctional liposome nanoparticle is a promising vector to deliver treatment agents to inner ear targets from a cochlear implant, where the spiral ganglion cells are a major concern. After intracochlear injection, distribution of nanoparticles in distal destinations, such as the semicircular canals, within one hour indicated that liposome nanoparticle can also be applied in drug delivery to the vestibular organ. The efficacy of delivery was enhanced by injecting a larger volume, likely due to the increased driving force, or by opening a second window into the scala vestibuli to avoid the pulsatile egress of cerebrospinal fluid (CSF) from the scala tympani. This reduced distribution with lower injection volumes suggests that diffusion of the hydrophobic liposome nanoparticles in the hydrophilic perilymph may be problematic. In the clinic practice of cochlear implantation, the constant release of nanoparticles from an advanced drug delivery electrode could resolve this diffusion issue.

Application of therapy into the middle ear, as a minimally invasive technique for inner ear drug delivery, is a favorable approach for the treatment of inner ear diseases that are not suitable for cochlear implantation. The outcome of intratympanic treatment would depend in a large part upon the efficacy of transportation from the middle ear to the inner ear. The existence of at least two pathways, including the round window membrane and the oval window, from the rat middle ear to the inner ear were recently supported by an *in vivo *MRI study [11]. There are a number of additional studies concerning round window membrane passage in the literature. The round window membrane appears well adapted for active transport. There are microvilli on the surface of outer epithelium and abundant organelles, such as mitochondria, rough endoplasmic reticulum, and a well-developed Golgi complex, that provide metabolic support and mechanisms for transport activity [14]. Our own unpublished data showed the appearance of clathrin in the round window membrane of rats, which suggests a specific pinocytosis. There is discontinuity in the basement membrane of the inner epithelium and loose junctions between inner epithelial cells that provide potential openings to support diffusion.. Both the surface characteristics and the size of the nanoparticles are important factors that affect the passage from the middle ear to the inner ear. One μm latex microspheres were reported to pass through the round window membranes of chinchillas and Rhesus monkeys [15]. In that study, the quantity of latex microspheres that passed through the round window membrane was unknown, but this is an important factor to define in studies of therapy directed toward inner ear diseases. In the present observations, liposome Gd nanoparticles of 130 nm passed the middle ear-perilymph barriers in quantities sufficient for MR imaging in 3 h and reached a signal intensity of above 1/5 of that in the middle ear cavity. Our preliminary histological study demonstrated augmented distribution of LPS+Gd-DOTA into the utricle of the vestibule than that in the spiral ligament capillary and spiral ganglion cells of the cochlea at 8 h following transtympanic injection of TRITC labeled LPS+Gd-DOTA in specimens taken from the rat after MRI study (data not shown). This implies the involvement of oval window in transporting LPS+Gd-DOTA. The oval window pathway was proved by abolish of immediate Gd-DOTA uptake in the vestibule and scala vestibuli induced by oval window sealing (data not shown). Potential uptake of TRITC labeled LPS+Gd-DOTA in other organs such as the brain, liver, and kidney should be investigated in a future study. These same advanced MFNPs showed targetability of their distribution in rat cochleae when functionalized with TrkB ligand [4] after intratympanic administration.

## Conclusions

In the present study, novel MRI visible multifunctional liposome nanoparticles were designed by encapsulating Gd-DOTA inside the hydrophilic core of the nanoparticles. Acceptable r1 values were realized in the final products. The Gd-DOTA-tagged liposome nanoparticles were visualized in the rat inner ear *in vivo *after both intracochlear and transtympanic injections. The intracochlear approach induced stronger signals on T1-weighted images. MRI also proved to be an excellent tool to monitor the purity of the Gd-DOTA-tagged liposome nanoparticles. There are potentially broad applications for these novel types of nanoparticles in future biomedical investigations.

## Methods

### Reagents and animals

Sphingosine (Sph), egg phosphatidylcholine (EggPC), and 1, 2-distearoyl-*sn*-glycero-3-phosphoethanolamine-N-[methoxy(polyethyleneglycol)-2000] (ammonium salt) [DSPE-PEG2000], 1,2-ditetradecanoyl-*sn*-glycero-3-phosphoethanolamine-N-diethylenetriaminepentaacetic acid (gadolinium salt) [DMPE-DTPA (Gd)], were from Avanti polar lipids (Alabaster, AL). Gd-DOTA (DOTAREM) was from Guerbet, Cedex, France. Hepes, and EDTA, were from Sigma. The purity of lipids was checked by thin-layer chromatography on silicic acid coated plate (Merck, Darmstadt, Germany) developed with a chloroform/methanol/water mixture (65:25:4, v/v/v). Examination of the plates after iodine staining, and when appropriate, upon UV illumination revealed no impurities. Lipid concentrations were determined gravimetrically with a high precision electrobalance (Cahn, Cerritos, CA). The other chemicals were of analytical grade and from standard sources.

Twenty-six male Wister rats, weighing between 270 g and 440 g, were provided by the Experimental MRI Laboratory, Department of Neurology, Helsinki University Central Hospital, FIN-00029 HUS, Helsinki, Finland. All animal experiments were approved by the Ethical Committee of the University of Tampere (permission: LSLH-2006-4143/Ym23). Animal care and experimental procedures were conducted in accordance with European legislation. Animals were divided into 3 groups: transtympanic injection of LPS+Gd-DOTA (TTI), intracochlear administration of LPS+Gd-DOTA with open perilymph circulation (IC-OPC), and intracochlear administration of LPS+Gd-DOTA with close perilymph circulation (IC-PPC) (Table 2). In TTI group, 2 rats received injection of unpurified LPS+Gd-DOTA and 12 rats were injected purified LPS+Gd-DOTA. All experimental procedures were performed under general anaesthesia, induced by intraperitoneal injections of medetomidine hydrochloride (0.5 mg/kg, Domitor, Orion, Finland) and ketamine (75 mg/kg, Ketalar, Pfizer, UK) and maintained at half dosage of the agents with the animal's eyes protected by Viscotears^® ^(Novartis healthcare A/S, Danmark).

**Table 2:** Grouping of animals and time of MRI measurement post-LPS+Gd-DOTA administration

Groups	N*	MRI time post-LPS+Gd-DOTA administration
TTI		
Unpurified	2	40 min, 90 min
purified	12	40 min, 90 min, 3 h, 6 h, 24 h, 48 h
IC-OPC	6	1 h
IC-CPC	5	0 min, 20 min, 30 min, 40 min, 1 h

*One positive control animal receiving transtympanic injection of Gd-DOTA (100 mM) was not counted in the numbers. IC-OPC: intracochlear administration with open perilymph circulation; IC-CPC: intracochlear administration with closed perilymph circulation; TTI: transtympanic injection.

### Preparation of liposomes containing Gd-DTPA-DMPE (LPS-DTPA-Gd)

**Figure 10:**
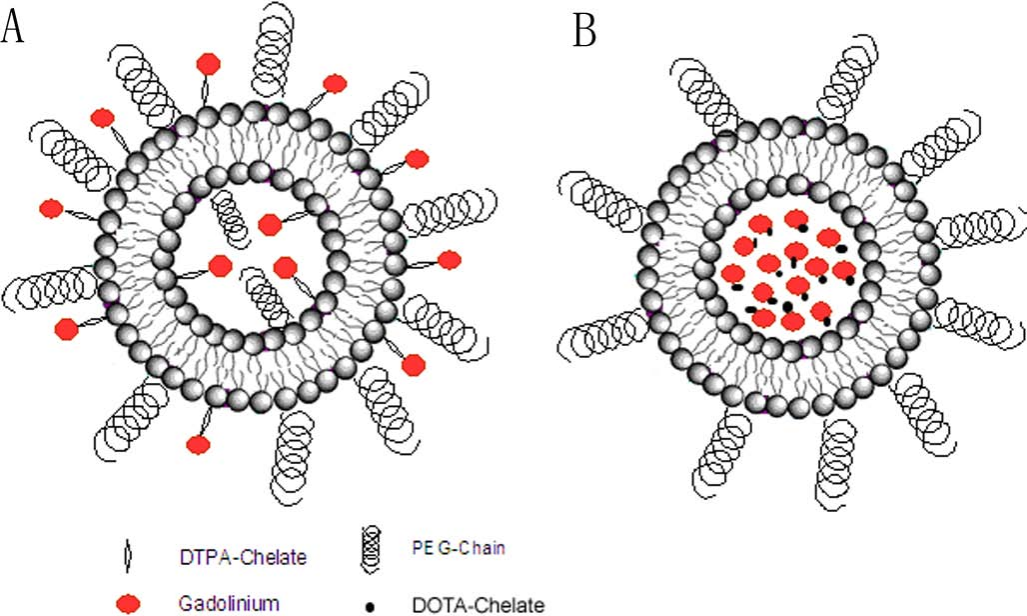
**Schematic representation of liposomes containing gadolinium chelate lipid (LPS-DTPA-Gd) (and liposomes encapsulating Gd-DOTA (LPS+Gd-DOTA)**. A: LPS-DTPA-Gd; B: LPS+Gd-DOTA.

Appropriate lipids along with DMPE-DTPA (Gd) (Gd chelate lipid) were mixed and solvent was evaporated under nitrogen to obtain a thin lipid film which was subsequently dried overnight under nitrogen. Lipid film was hydrated with buffer and resulting MLV's were then sequentially extruded 400 (five times), 200 (five times), and 100 nm (seven times) polycarbonate Millipore filters to yield unilamellar liposomes containing gadolinium in both leaflets (Figure 10A). The size of LPS-DTPA-Gd was 130 ± 20 nm.

### Preparation of liposomes encapsulating Gd-DOTA (LPS+Gd-DOTA)

**Figure 11:**
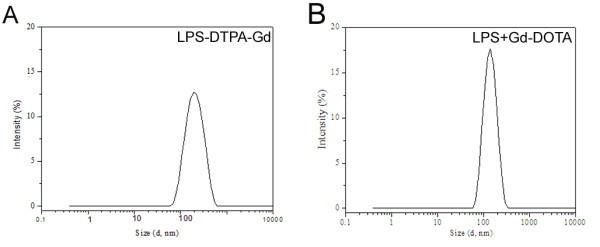
**Size distribution by intensity of purified liposomes (total lipid concentration 1 mM) containing DTPA-Gd (LPS-DTPA-Gd) or encapsulating Gd-DOTA (LPS+Gd-DOTA) obtained by dynamic light scattering**. Temperature was maintained at 25°C. LPS-DTPA-Gd (A) showed more dispersed size distribution than LPS+Gd-DOTA (B).

Lipids were dissolved in chloroform and subsequently mixed in this solvent so as to obtain the desired compositions. The solvent was removed using a gentle stream of nitrogen whereafter the dry lipid residues were maintained under reduced pressure for at least 24 h to remove trace amounts of chloroform. The lipid film was hydrated with Gd-DOTA solution (500 mM, Guerbet, Cedex, France) at 60°C for 60 min. The resulting lipid suspensions were extruded (Avestin, Ottawa, Canada) through 400 (five times), 200 (five times), and 100 nm (seven times) polycarbonate Millipore filters to yield large unilamellar vesicles (LUV's) containing Gd-DOTA both inside as well as outside of the liposomes (Figure 10B). After extrusion, external gadolinium was removed by Sephadex G-50 (fine) quick spin columns (Roche Diagnostics GmbH, Mannheim, Germany). Before and after removal of the external Gd-DOTA average particle size (Z_av_, using DLS, Zetasizer Nano ZS, Malvern Instruments Ltd., UK) of 300 ± 20 and 130 ± 20 nm was obtained (Figure 11). All LPS+Gd-DOTA nanoparticles contain 500 mM Gd-DOTA. Liposomes containing Gd-DOTA were stable even after one week of preparation as confirmed by DLS, revealing no changes in Z_av_.

### Phantom study

A 4.7 T MR scanner with bore diameter of 155 mm (PharmaScan, Bruker BioSpin, Germany) was used in both phantom and in vivo MR measurements. The maximum gradient strength was 300 mT/m with an 80-μs rise time. A dedicated rodent head coil (linear bird cage coil) with diameter of 38 mm was used for the phantom and animal studies. To evaluate T1 and T2 relaxation times of *LPS-DTPA-Gd *nanoparticles and analyze the contribution of water-accessible Gd-DTPA to MR signal, the nanoparticle solutions were prepared with physiological saline at a concentration of (1 mM containing 0.5 mM Gd). 5% SDS was applied to disassemble the nanoparticles and expose all the lipid-bond Gd-DTPA to the water. This was done to detect MRI signal from all of the Gd-DTPA associated with the nanoparticles, including that amount within the nanoparticles that could be shielded by the lipid from proton interaction. Gd-DOTA diluted with physiological saline at a concentration of 0.5 mM was used as a positive control. Plastic phantom tubes (400 μl, AgnTho's AB, Sweden) were filled with the solutions of Gd-DOTA, *LPS-DTPA-Gd *nanoparticles, or *LPS-DTPA-Gd *nanoparticles plus 5% SDS.

In order to measure relaxivities, r1 and r2, of LPS+Gd-DOTA, solutions of various concentrations were placed into plastic phantom tubes arranged concentrically within a 50 ml syringe. Dilutions were made with artificial perilymph containing 145.5 mM NaCl, 2.7 mM KCl, 2.0 mM MgSO4, 1.2 mM CaCl2, 5.0 mM HEPES, pH adjusted to 7.4 [16]. Negative controls were prepared using either plain artificial perilymph or HEPES buffer. Each sample was prepared in duplicate.

T2 relaxation time was determined using a multi-slice multi-echo sequence (MSME), based on CPMG (Carr-Purcell Meiboom-Gill) spin echo (SE) [repetition time (TR) 1500 ms, echo time (TE) 7-229 ms, 32 echo times, matrix size 128 × 128, single slice, slice thickness 2.0 mm, field of view (FOV) 5.0 cm, resolution 0.098 × 0.130 mm2, number of excitation (NEX) 3]. T1 relaxation times were determined by rapid acquisition with relaxation enhancement (RARE) sequence with variable TR (TR 100, 432, 859, 1458, 2472, 7500 ms, TEeff 8.7 ms, RARE factor 2, NEX 1, matrix size 128 × 128, FOV 5 cm, single slice with slice thickness 2.0 mm).

### Transtympanic injection of LPS+Gd-DOTA

LPS+Gd-DOTA nanoparticles were administered through transtympanic injection under an operating microscope as previously reported [11]. An incision of approximately 1 mm length was made in the anterior superior quadrant of the tympanic membrane by a 25 gauge needle to release any potential air bubble. Either purified or unpurified 0.04 ml LPS+Gd-DOTA (1 mM LPS+Gd-DOTA containing 500 mM Gd-DOTA) were injected into the middle ear cavity through the posterior superior quadrant of the tympanic membrane. As a positive control, Gd-DOTA (100 mM) was transtympanically injected as above.

### Intracochlear administration of LPS+Gd-DOTA to open perilymphatic compartments

The bulla was exposed through a post-auricular approach. Working under the operating microscope, a hole was drilled through the bulla with a 2 mm diameter burr. The round window membrane was identified superior to the stapedial artery as reported previously [1]. The scala tympani of the basal turn was opened with a 0.5 mm cutting burr inferior to the stapedial artery and the round window. A second opening was made in the scala vestibuli of the basal turn. After wicking away the perilymph that egressed through the opening, two pieces of gelatin sponge soaked in LPS+Gd-DOTA (the size of each piece was proximately 3 mm^3 ^after soaking in the LPS+Gd-DOTA solution) were placed against the openings in the middle ear cavity. The bulla opening was covered by muscle and the wound was sutured closed.

### Intracochlear injection of LPS+Gd-DOTA into closed perilympatic compartments

After drilling into the scala tympani of the basal turn as above, polyimide tubing (MicroLumen Inc., Tampa, USA) was inserted intraluminally into a polyethylene tubing (I.D. 0.28 mm, O.D. 0.61 mm, Becton Dickinson, Franklin Lakes, USA) and fixed with nail polish. The tubing was filled with LPS+Gd-DOTA solution. After opening the scala tympani of the basal turn with a 0.5 mm cutting burr, the high performance polyimide tubing tip (MicroLumen, Tampa, USA), which was connected to polyethylene tubing (I.D. 0.28 mm, O.D. 0.61 mm, Becton Dickinson and Company, USA) and primed with LPS+Gd-DOTA solution was inserted into the scala tympani and was sealed circumferentially with Histoacryl (enbucrilate) glue (Aesculap AG, Tuttlingen, Germany). LPS+Gd-DOTA nanoparticles were initially applied by bolus injection of 5 μl, but they failed to demonstrated any MRI detectable signal for uncertain reasons. Although it is a physiologically excessive volume, 50 μl was injected and produced satisfying MR signal. The remaining observations were performed with 50 μl LPS+Gd-DOTA. The bulla opening was covered by muscle and the wound was sutured closed.

### In vivo MRI observation of LPS+Gd-DOTA in the inner ear

The body temperatures of the rats were maintained by circulating warm water and their respirations were recorded with Physio Tool-1.0.b.2 program (Bruker, Germany). Rats were placed in the magnet with the ears positioned at the isocenter. 2D and 3D MRI measurement was performed. T1-weighted 2D images were acquired with RARE sequence (TR/TEeff 500/10 ms, RARE factor 4, matrix size 256 × 192, slice thickness 0.5 mm, FOV 2.5 × 2.5 cm2, resolution 0.098 × 0.13 mm2, NEX 33). T1-weighted 3D images were acquired with RARE sequence (TR/TEeff 500/12 ms, RARE factor 16, matrix size 64 × 64 × 64, FOV 0.89 × 0.89 × 0.89 cm3, resolution 0.139 × 0.139 × 0.139 mm3, NEX 2).

For the groups who had either intracochlear administration of LPS+Gd-DOTA with open perilymph circulation or transtympanic injection of LPS+Gd-DOTA, MRI scanning commenced immediately after the procedure. After setting up the geometry with T2-weighted 2D imaging, T1-weighted 2D, and T1-weighted 3D images were acquired at different time points. In the group of intracochlear injection of LPS+Gd-DOTA with closed perilymph circulation, LPS+Gd-DOTA nanoparticles were injected after setting up the MRI geometry. Then T1-weighted 2D, and T1-weighted 3D images were acquired as above. MRI measurements were followed up to 48 h in the group of transtympanic injection of LPS+Gd-DOTA and for one hour in the group of intracochlear administration of LPS+Gd-DOTA with open perilymph circulation (Table 2).

### 3D volume rendering of the inner ear MRI

OsiriX v3.3.2 (OsiriX Foundation, Geneva, Switzerland) software was applied for 3D rendering of the 3D raw stacked MR images of the inner ear. 3D volume renderings were created for visualisation of the inner ear structures with the ability to rotate the images into various orientations and to create photographs.

### Quantification of signal intensity and statistics

ParaVision PV 4.0 (Bruker, Germany) software was used for post-processing of MR images and calculations of T1 and T2 relaxation times in phantom and signal intensity measurements in the inner ear compartments. Signal intensities in the region of interest were measured in the middle ear cavity, the vestibule, the scala vestibuli and scala tympani in the higher basal turn, and the brainstem using ParaVision 4.0. Differences in the signal intensities between 3 h and 6 h time points among these compartments were compared with student t-test. Signal intensity ratio of the vestibule, the scala vestibuli, and scala tympani over the middle ear was defined as the indicator of transport efficacy and compared between LPS+Gd-DOTA and Gd-DOTA groups with student t-test. A difference was considered to be statistically significant at P < 0.05. Photoshop CS3 software was used for labeling and demonstration of the inner ear anatomy in MR image.

## Competing interests

The authors declare that they have no competing interests.

## Authors' contributions

JZ participated in the design of the study and performed the MRI measurement. RS and SR participated to the design of the liposomes and prepared the liposomes. DP participated in MRI measurements. UAR was responsible for the sequences of MRI. PK supervised the design and preparation of liposomes. IP supervised the study. All authors have read and approved the final manuscript.

## References

[B1] Zou J Zhang W Poe D Qin J Fornara A Zhang Y Ramadan UA Muhammed M Pyykko I MRI manifestation of novel superparamagnetic iron oxide nanoparticles in the rat inner ear Nanomedicine (Lond) 5 739 754 10.2217/nnm.10.45 20662645

[B2] Nanoear: 3g-Nanotechnology based targeted drug delivery using the inner ear as a model target organ http://www.nanoear.org/

[B3] Zou J Saulnier P Perrier T Zhang Y Manninen T Toppila E Pyykko I Distribution of lipid nanocapsules in different cochlear cell populations after round window membrane permeation J Biomed Mater Res B Appl Biomater 2008 87 10 18 18437698 10.1002/jbm.b.31058

[B4] Zou J Zhang Y Zhang W Ranjan S Sood R Mikhailov A Kinnunen P Pyykko I Internalization of liposome nanoparticles functionalized with TrkB ligand in rat cochlear cell populations European Journal of Nenomedicine 2009 3 8 14

[B5] Ishii T The fine structure of lipofuscin in the human inner ear Arch Otorhinolaryngol 1977 215 213 221 10.1007/BF00463059 195564

[B6] Walther LE Westhofen M Presbyvertigo-aging of otoconia and vestibular sensory cells J Vestib Res 2007 17 89 92 18413901

[B7] Bohne BA Gruner MM Harding GW Morphological correlates of aging in the chinchilla cochlea Hear Res 1990 48 79 91 10.1016/0378-5955(90)90200-9 2249962

[B8] Horner KC Guilhaume A Ultrastructural changes in the hydropic cochlea of the guinea-pig Eur J Neurosci 1995 7 1305 1312 10.1111/j.1460-9568.1995.tb01121.x 7582104

[B9] Igarashi Y Ishii T Lipofuscin pigments in the spiral ganglion of the rat Eur Arch Otorhinolaryngol 1990 247 189 193 10.1007/BF00176535 2350511

[B10] Zhang Y Zhang W Johnston AH Newman TA Pyykko I Zou J Improving the visualization of fluorescently tagged nanoparticles and fluorophore-labeled molecular probes by treatment with CuSO(4) to quench autofluorescence in the rat inner ear Hear Res 2010 269 1 11 10.1016/j.heares.2010.07.006 20659540

[B11] Zou J Ramadan UA Pyykko I Gadolinium uptake in the rat inner ear perilymph evaluated with 4.7 T MRI: a comparison between transtympanic injection and gelatin sponge-based diffusion through the round window membrane Otol Neurotol 2010 31 637 641 20142794 10.1097/MAO.0b013e3181d2f095

[B12] Counter SA Bjelke B Klason T Chen Z Borg E Magnetic resonance imaging of the cochlea, spiral ganglia and eighth nerve of the guinea pig Neuroreport 1999 10 473 479 10.1097/00001756-199902250-00006 10208574

[B13] Bousquet JC Saini S Stark DD Hahn PF Nigam M Wittenberg J Ferrucci JT Jr Gd-DOTA: characterization of a new paramagnetic complex Radiology 1988 166 693 698 3340763 10.1148/radiology.166.3.3340763

[B14] Goycoolea MV Clinical aspects of round window membrane permeability under normal and pathological conditions Acta Otolaryngol 2001 121 437 447 10.1080/000164801300366552 11508501

[B15] Goycoolea MV Muchow D Schachern P Experimental studies on round window structure: function and permeability Laryngoscope 1988 98 1 20 10.1288/00005537-198806001-00002 3287079

[B16] Takemura K Komeda M Yagi M Himeno C Izumikawa M Doi T Kuriyama H Miller JM Yamashita T Direct inner ear infusion of dexamethasone attenuates noise-induced trauma in guinea pig Hear Res 2004 196 58 68 10.1016/j.heares.2004.06.003 15464302

